# Beyond Infection: Unmasking the Impact of COVID-19 on Diabetes Emergency Trends

**DOI:** 10.7759/cureus.68566

**Published:** 2024-09-03

**Authors:** Sedat C Güney, Can Akçura, Samet Alkan, Gamze Gelir Çavdar, Nilüfer Özdemir, Zeliha Hekimsoy

**Affiliations:** 1 Endocrinology and Metabolism, Manisa Celal Bayar University, Manisa, TUR; 2 Endocrinology and Metabolism, İzmir City Hospital, İzmir, TUR

**Keywords:** coronavirus pandemic, endocrinology consultations, hypoglycemia, diabetic ketoasidosis, diabetic emergencies, diabetes mellitus, covid-19

## Abstract

Introduction

The relationship between COVID-19 and diabetes has been demonstrated in many studies. However, it is thought that the psycho-socioeconomic effects of the pandemic led to a worsening of glycemic control and an increase in diabetes-associated clinical emergencies in diabetic patients without a diagnosis of COVID-19.

Objectives

We aimed to reveal the change in the frequency of diabetes-related clinical emergencies before and during the COVID-19 pandemic.

Patients and methods

The data of the patients requiring endocrinology consultations in Manisa Celal Bayar University Faculty of Medicine Emergency Service between March 2018 and March 2022 were included. In total, 269 consultations were analyzed. The March 2018 - March 2020 period was considered as pre-COVID, and March 2020 - March 2022 as the COVID-19 period. The frequency of diabetes-related conditions between these two periods was compared.

Results

Compared to the pre-COVID period, there was a significant increase in the frequency of admissions with diabetic ketosis, hyperglycemic hyperosmolar state, hypoglycemia, and hyperglycemia in the COVID-19 period (p=0.022, p=0.037, p=0.044, and p=0.004 respectively). Although an increase was observed in the frequency of diabetic ketoacidosis (DKA) cases, no statistical significance was found. When the mortality data of the patients was evaluated, the total number of deaths was seen to increase significantly in the COVID-19 period (p=0.01). It was observed that the ratio of type 2 diabetes mellitus (DM)/type 1 DM among DKA patients increased significantly in the COVID-19 period (p=0.001).

Conclusions

It can be concluded that the increasing trend in diabetic emergencies that started even before the pandemic is exacerbated by COVID-19, especially in patients with poor glycemic control.

## Introduction

The COVID-19 disease, which was caused by the severe acute respiratory syndrome coronavirus 2 (SARS-CoV-2), was first identified in Wuhan, China in December 2019 [1]. It was declared a pandemic by the World Health Organization on March 11, 2020, which is also when the first case was reported in Türkiye.

Advanced age, socioeconomic deprivation, male gender, non-white ethnicity, presence of diabetes mellitus (DM), and other chronic diseases have been identified as risk factors for poor outcomes in patients with COVID-19 [2]. Moderate hyperglycemia is also common in patients with COVID-19 and is associated with worse outcomes, even in individuals without diabetes [3]. The relationship between COVID-19 and diabetes has been clearly demonstrated in most of the studies. However, it is thought that the psycho-socioeconomic effects of the pandemic lead to the worsening of glycemic control and an increase in diabetes-associated clinical emergencies in diabetic patients without a diagnosis of COVID-19 [4, 5].

Although there are conflicting studies, most studies have shown that COVID-19 causes an increased risk of diabetic ketoacidosis (DKA) and is associated with increased mortality in individuals with DKA [6-10]. The increase in glucagon and cortisol levels associated with the stress response to COVID-19 can lead to relative insulin deficiency, which may contribute to the development of diabetic ketoacidosis (DKA), especially in diabetic patients who do not receive insulin treatment [11-13]. A systematic review and meta-analysis revealed that COVID-19 leads to higher levels of serum cortisol, and this increase is linked with the severity of the COVID-19 infection [14]. It is conceivable that the frequency of other diabetic emergencies may have increased due to similar pathogenetic mechanisms. To the best of our knowledge, the frequency of diabetes-related events other than DKA during the COVID-19 pandemic period has not been investigated before. In this study, we analyzed the change in the frequency of diabetes-related clinical emergencies before and during the COVID-19 pandemic.

## Materials and methods

Study participants and protocol

In this retrospective study, data of the patients requiring endocrinology consultations in Manisa Celal Bayar University Faculty of Medicine Emergency Service between March 2018 and March 2022 were obtained from the hospital database system.

Inclusion criteria

All patients aged 18 or above, who received endocrinology consultations at Manisa Celal Bayar University Faculty of Medicine Emergency Service between March 2018 and March 2022. Consultations specifically related to diabetes emergencies, as this forms the core focus of the study's final analysis.

Exclusion criteria

Repeated consultations for the same patients within the study period were excluded to avoid duplication and to ensure each consultation was counted as a unique event. This was achieved by reviewing patient identifiers and consultation dates in the hospital database, ensuring that only the first consultation for each patient within the study period was included. Consultations that did not relate to diabetes emergencies were excluded from the final analysis to maintain the focus on the study's primary objective, which is the assessment of diabetes-related conditions and emergencies.

**Figure 1:**
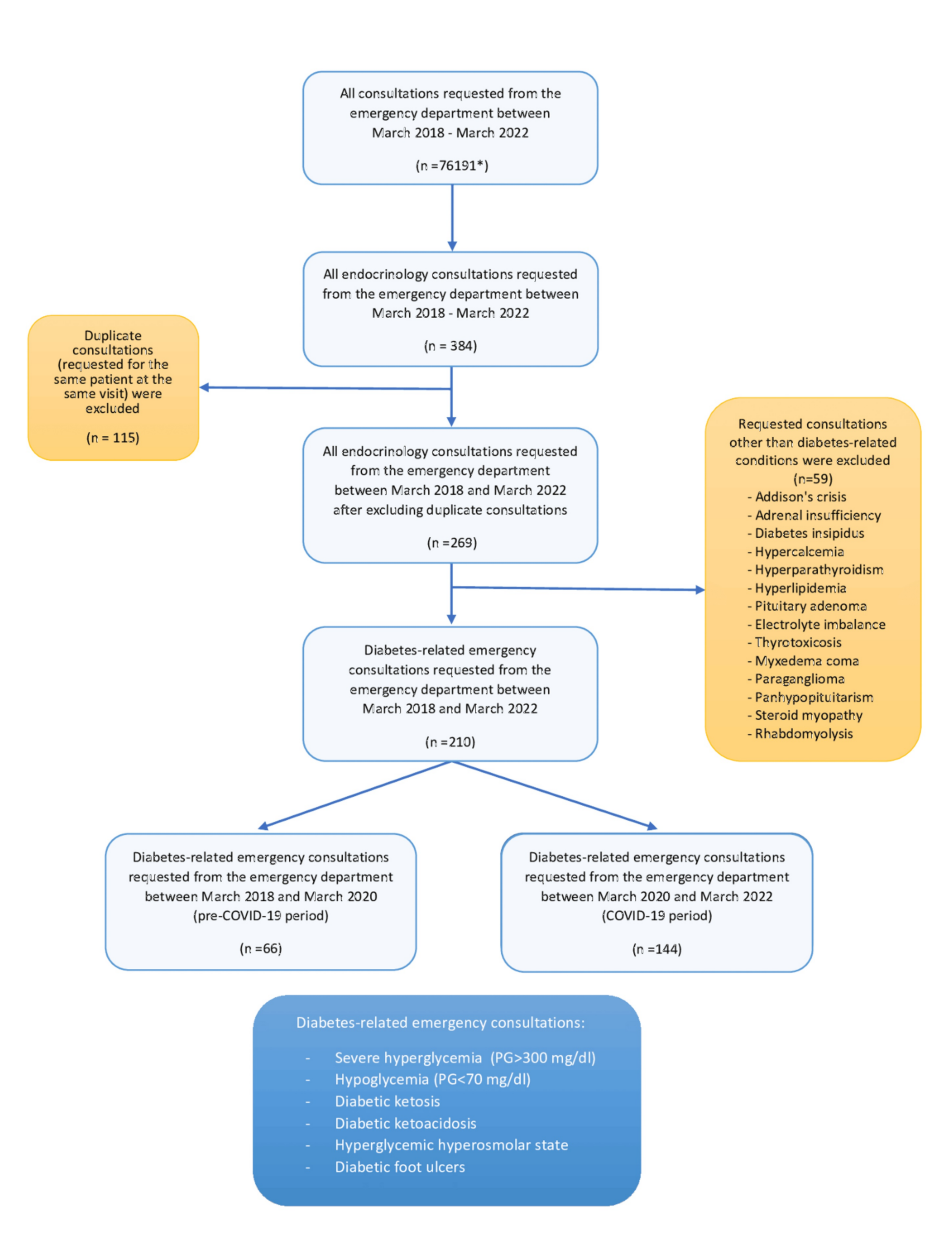
Flow diagram of the consultation data identification and selection process * n=33,254 (in pre-COVID-19 period) vs n=42,937 (in COVID-19 period), total=76,191

Excluding the repeated consultations of the same patients left 269 consultations to be analyzed. Of these 269 consultations, it was seen that 210 were requested due to diabetes-related emergencies. Thus, the final analysis was conducted based on these 210 consultations (Figure 1). The reasons for admission, consultation duration, demographic data, post-consultation status, diagnoses, and laboratory findings of the patients were recorded. The period between March 2018 and March 2020 was considered pre-COVID, and the period between March 2020 and March 2022 was the COVID-19 period. The frequency of diabetes-related conditions between these two periods was compared. The study was approved by the Ethics committee of Manisa Celal Bayar University Faculty of Medicine on 08/03/2023 (approval number 20.478.486)

Definitions of diabetic emergencies

In our center, all patients who apply to the emergency department for any reason and are diagnosed with a diabetes-related emergency are consulted with the endocrinology department. The conditions we classify as diabetes-related emergencies include severe hyperglycemia (BG >300 mg/dl), hypoglycemia (BG <70 mg/dl), diabetic ketosis or ketoacidosis, diabetic foot infection, and hyperglycemic hyperosmolar state. Diabetic ketoacidosis was diagnosed when plasma glucose level was >250 mg/dl, ketone ≥1.5 mmol in urine (with nitroprusside method), and blood pH ≤7.30 or serum bicarbonate (HCO3-) level ≤15 mEq/l. In the absence of acidosis or low bicarbonate levels despite the presence of ketonuria, the case was considered diabetic ketosis. Criteria for the hyperglycemic hyperosmolar state are the following: marked elevations in blood glucose levels (≥600 mg/dL) and in serum osmolarity (≥320 mOsm/L), with a pH level greater than 7.30 and mild or absent ketosis.

Statistical analysis

The concordance of the quantitative data to normal distribution was examined using the Kolmogorov-Smirnov test. In the comparison of normally distributed variables between groups, an independent samples t-test was used, and descriptive statistics were shown as mean ± standard deviation. In the analysis of qualitative variables, Chi-squared tests were used according to the groups, and the results were presented in the form of frequency (%). Results were considered statistically significant when p<0.05.

## Results

Diabetes and diabetes-related clinical emergencies accounted for 78% (n=210) of the endocrinology consultations requested from the emergency department. The data obtained from these 210 patients was analyzed. The mean age of the patients was 52.7 ± 19.9 years. 51.9% (n=109) of the patients were female and 48.1% (n=101) were male. The mean response time to consultations was 67.8 ± 41.5 minutes.

Compared to the pre-COVID period, there was a significant increase in the frequency of admissions with diabetic ketosis, hyperglycemic hyperosmolar state (HHS), hypoglycemia, and hyperglycemia in the COVID-19 period. Although an increase was observed in the frequency of DKA cases, no statistical significance was found (Table 1).

**Table 1:** Diabetes-related clinical emergencies pre-COVID-19 and COVID-19 period * Chi-square analysis; ** p<0.05 significant difference; Frequencies described as number (n) and percentage (%)

Diagnosis	Pre-COVID-19 period n (%)	COVID-19 period n (%)	Total n (%)	*p-value
Diabetic ketoacidosis	32 (40%)	48 (60%)	80 (100%)	0.250
Hyperglycemic hyperosmolar state	1 (12.5%)	7 (87.5%)	8 (100%)	**0.037
Diabetic ketosis	13 (28.9%)	32 (71.1%)	45 (100%)	**0.022
Hypoglycemia	2 (18.2%)	9 (81.2%)	11 (100%)	**0.044
Hyperglycemia	16 (26.7%)	44 (73.3%)	60 (100%)	**0.004
Diabetic foot	2 (33.3%)	4 (66.7%)	6 (100%)	0.300
Total	66 (31.4%)	144 (68.6%)	210 (100%)	**0.001

While the number of consultations requested from the emergency department for all units was 33.254 in the pre-COVID period, it was observed as 42.937 in the COVID-19 period. The number of endocrinology consultations is seen to increase gradually every year. Diabetes-related endocrinology consultations were 0.2% of all consultations (n=66) in the pre-COVID period and 0.34% (n=144) in the COVID-19 period (p=0.001). Among the 144 patients in the COVID-19 era, 31 were diagnosed with COVID-19 (24 serologically tested positive for COVID-19, while seven patients tested negative for COVID-19 but had clinical and radiological findings consistent with COVID-19 infection). DKA constituted the largest part (38.1%) of diabetes-related consultations. In the consultations evaluated, it was seen that 50.9% of the patients were hospitalized and followed up on, and 14.3% were referred to another center due to insufficient numbers of vacancies at our hospital.

When the mortality data of the patients was evaluated, the total number of deaths was seen to increase significantly in the COVID-19 period (n=15) compared to the pre-COVID period (n=3; p=0.01; Table 2). Sixty percent of the deaths in the COVID-19 period were directly related to COVID-19. The COVID-19 polymerase chain reaction (PCR) test result was positive in seven mortalities in the COVID-19 period (p=0.001). Although the PCR test result was negative in two patients, they were thought to be COVID-19, with the findings of a computed tomography of the thorax. Thus, 60% of the deaths in the COVID-19 period were directly related to COVID-19 (p=0.001).

**Table 2:** Mortality data of patients pre-COVID-19 and COVID-19 period *p=0.01 (Chi-squared analysis)

Diagnosis	Deaths		Total (n)
	Pre-COVID-19 period n (%)	COVID-19 period n (%)	
Diabetic ketoacidosis	1 (3.1%)	4 (8.3%)	5 (6.2%)
Hyperglycemic hyperosmolar state	0 (0%)	2 (28.5%)	2 (25%)
Diabetic ketosis	1 (7.7%)	4 (12.5%)	5 (11.1%)
Hypoglycemia	0 (0%)	3 (33.3%)	3 (27.2%)
Hyperglycemia	1 (6.2%)	1 (2.2%)	2 (3.3%)
Diabetic foot	0 (0%)	1 (25%)	1 (16.6%)
Total deaths*	3 (4.5%)	15 (10.4%)	18 (8.5%)

When the laboratory results of the DKA cases in the pre-COVID period and in the COVID-19 period were analyzed, no significant difference was found (Table 3).

**Table 3:** Mean values of laboratory data in diabetic ketoacidosis cases before and during COVID-19 * Independent samples T test

Laboratory data	Pre-COVID-19 period	COVID-19 period	*p-value
pH	7.13	7.15	0.687
Bicarbonate (mmol/L)	11.1	10.8	0.817
Lactate (mmol/L)	3.13	3.16	0.945
Urine ketone (mmol/L)	2.9	2.6	0.285
Plasma glucose (mg/dl)	553	481	0.085
Serum Na (mEq/L)	134	132	0.107
Serum K+ (mEq/L)	4.4	4.4	0.809
Serum creatinine (mg/dl)	1.07	1.29	0.364
Estimated glomerular filtration rate (ml/min/1.73m^2^)	81.7	81.3	0.954

**Figure 2:**
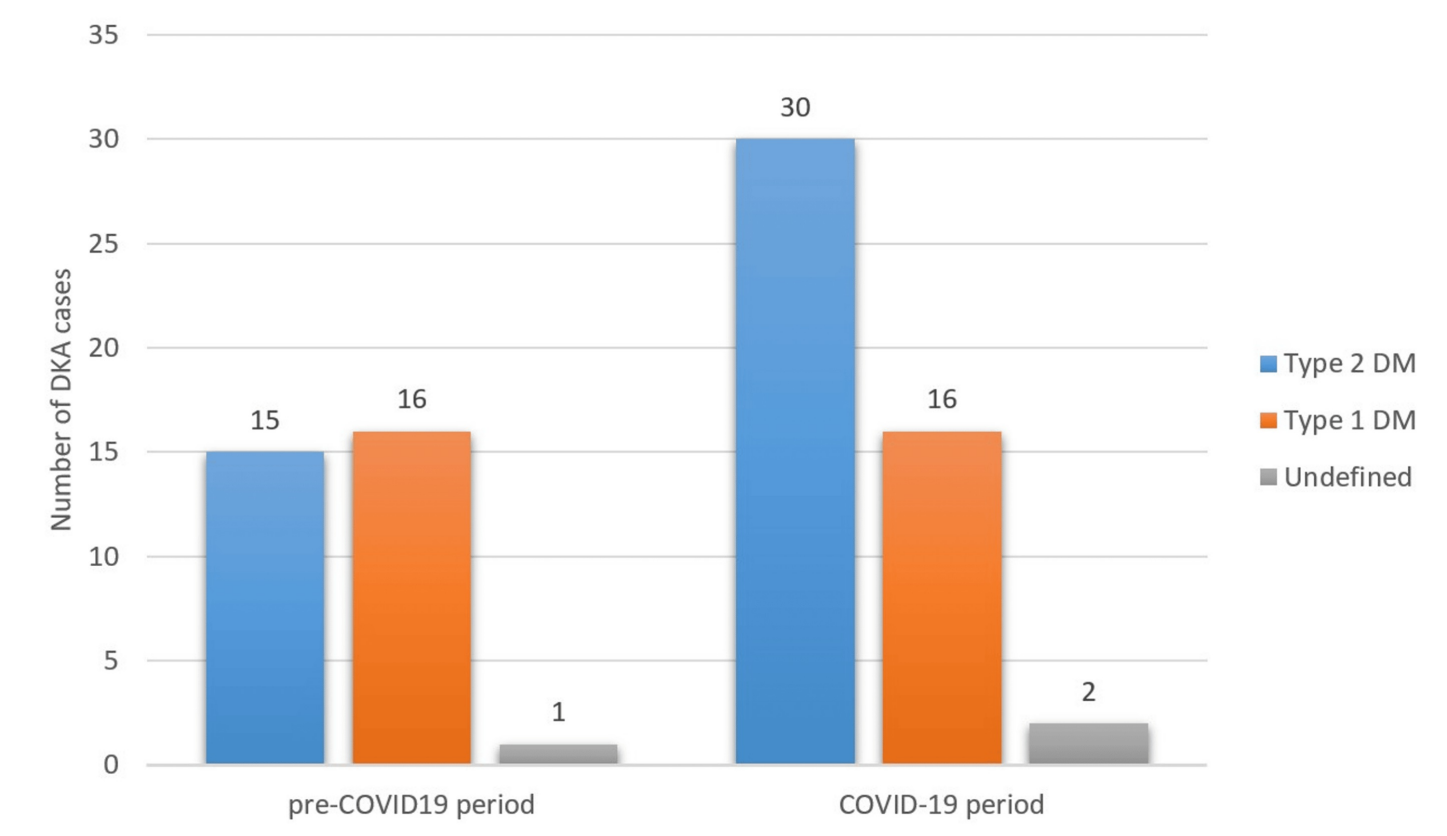
Distribution of patients presenting with DKA in the pre-COVID-19 and COVID-19 period by diabetes type DKA - diabetic ketoacidosis

**Figure 3:**
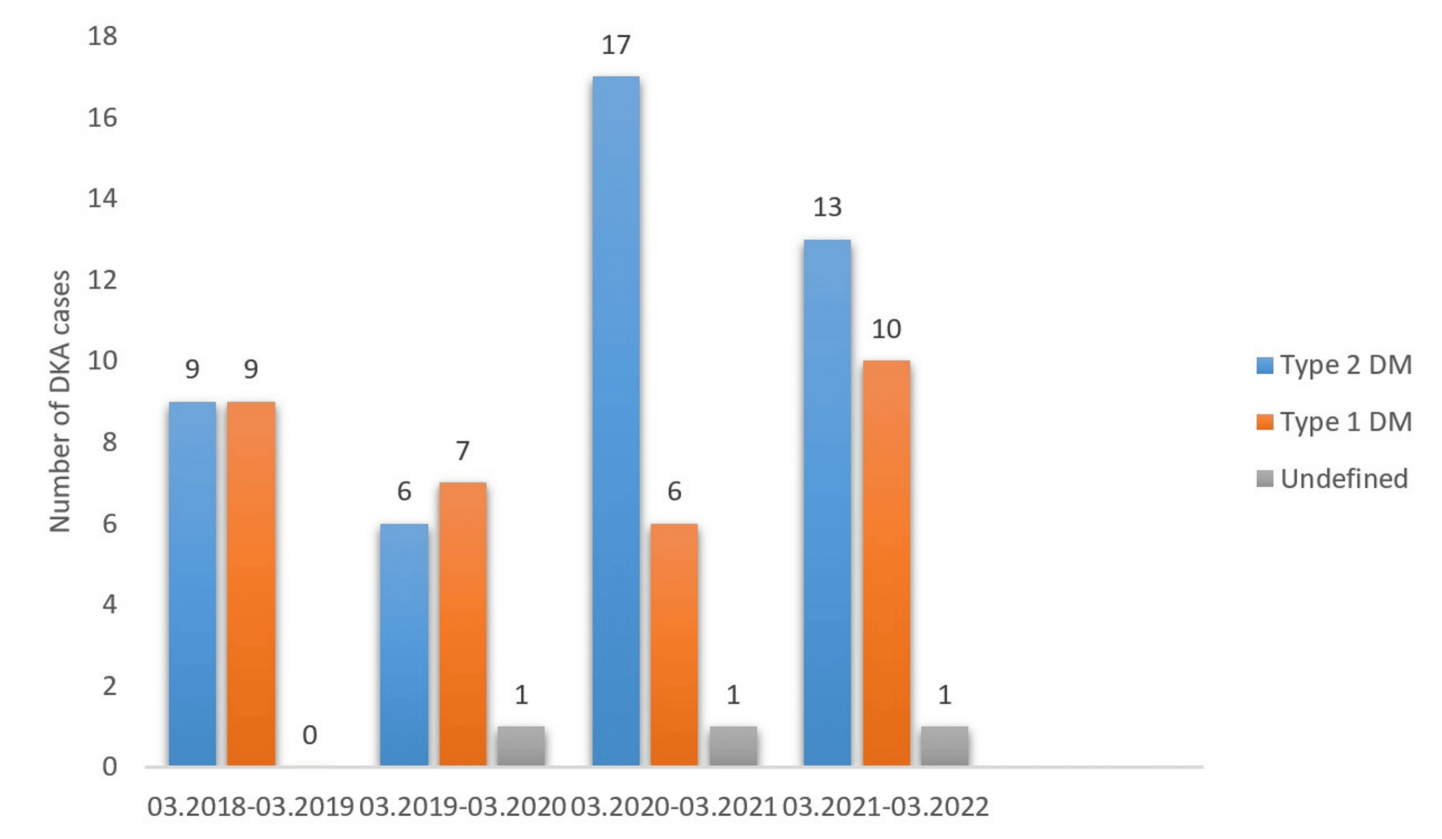
Annual distribution of patients presenting with DKA by diabetes type DKA - diabetic ketoacidosis

Considering the distribution of type 1 diabetes mellitus (DM) and type 2 DM patients present with diabetic ketoacidosis, type 2 DM patients among DKA patients were observed to increase significantly in the COVID-19 period (p=0.001). While the number of type 1 DM patients among admissions with DKA cases remained stable in the pre- and COVID-19 period, the number of cases with type 2 DM increased by 100%. This increasing trend in favor of type 2 DM was clearly detected in the first year of the COVID-19 pandemic, and it continued in the second year of the pandemic, although the rate of increase slowed slightly (Figures 2 and 3).

The frequency of admission to the emergency department with hypoglycemia increased significantly after the pandemic. Five out of 11 of the patients (45%) present with hypoglycemia were on gliclazide treatment, and four out of 11 (36.3%) were on insulin treatment. There was no patient using sulfonylurea and insulin together. Seven of the patients had severe hypoglycemia (plasma glucose level <54 mg/dL). While the mean glucose level of the hypoglycemia cases was 59 mg/dl in the pre-COVID period, it was found to be 49 mg/dl in the COVID-19 period (p=0.58). Among the hypoglycemia cases, three mortalities were recorded. Two of these mortalities were due to COVID-19, and the other one was due to drug use.

## Discussion

The possible causes of the negative effects of the COVID-19 pandemic on diabetes management may include the psycho-social effects of the pandemic, stress-induced hyperglycemia triggered by infection, dysfunction of pancreatic beta cells, and increased use of corticosteroids [3].

Psycho-social effects of the pandemic in individuals with pre-existing diabetes: deterioration of glycemic control due to increased obesity, decreased physical activity, and sedentary life in the pandemic, and less medical care due to social restrictions [15]. In a global study involving healthcare professionals from 47 countries, it was revealed that the chronic condition most affected by COVID-19 due to disruptions in follow-up was diabetes [16]. Patients with chronic diseases such as diabetes have avoided or postponed seeking medical attention for routine problems not related to COVID-19 out of fear of infection or to help reduce the burden on healthcare services already overloaded by COVID-19 [17]. Depressive mood and loss of motivation, along with restrictions on human movements during the pandemic, were also associated with an increase in physical inactivity and sedentary life [18]. It has been shown that the frequency of obesity increased during this period [19].

Infection-related stress hyperglycemia: relative insulin resistance secondary to increased glucagon and cortisol levels due to the stress response triggered by acute severe conditions such as severe infections or myocardial infarction, increased free fatty acid secretion, increased lipolysis, increased circulating inflammatory mediators, increased gluconeogenesis [3, 20]. Stress hyperglycemia may be even more severe due to cytokine storm in COVID-19 [13].

Beta-cell dysfunction occurs as a result of the binding of the SARS-CoV-2 virus to ACE-2 receptors in pancreatic beta cells [21]. SARS-CoV2 virus binds to ACE-2 receptor, causing a decrease in ACE-2 level and thus activation of the renin-angiotensin system. This can initiate cell dysfunction and inflammation, leading to insulin resistance [13]. 

With the widespread use of steroids for COVID-19 treatment, normoglycemia is thought to have become more difficult to maintain [22].

In our study, we found that there was a statistically significant increase in the frequency of admissions to the emergency department with diabetic ketosis, hyperglycemic hyperosmolar state, hypoglycemia, and hyperglycemia after the COVID-19 pandemic. Although an increase was observed in DKA cases, it was not statistically significant.

Li et al., in their study of 658 hospitalized COVID-19 patients, suggested that a COVID-19 infection may lead to the development of ketosis or ketoacidosis even in non-diabetic patients and stated that this may be related to the acceleration of fat breakdown by COVID-19. They also showed that ketosis increases the length of hospitalization and mortality rate [8].

Ditkowsky et al. compared DKA cases admitted to the emergency department between March and May in the years 2019 and 2020 and stated that 106 (0.114%) of 93,218 emergency service admissions in 2019 and 214 (0.363%) of 59,009 admissions in 2020 were diagnosed with DKA. They pointed out that although the number of admissions decreased, there was an increase in the number of DKAs [9].

In our study, although the increase in the number of DKA cases during the pandemic period was not of statistical significance, a significant increase in the rate of type 2 diabetics among DKA cases was noted. This situation was similar to the study of Misra et al. [23], the most comprehensive study on this subject. In this study, while the number of admissions to the emergency department with DKA decreased in type 1 DM cases compared to the pre-COVID-19 period, a significant increase was observed in type 2 DM cases. In the same study, among the patients present with DKA, those with a diagnosis of COVID-19 had a higher in-hospital mortality rate than those without a diagnosis of COVID-19 (OR: 4.8 95% CI 4.3-5.3) [23]. In a study conducted in the pre-COVID-19 period, an increase in the frequency of DKA in type 2 DM cases was noted [24]. A worsening of glycemic control due to reasons such as obesity, decreased physical activity, sedentary lifestyle, less medical care due to social restrictions, relative insulin deficiency due to infection-related stress response, and hyperglycemia due to the widespread use of steroids may be responsible for the increase in DKA frequency in type 2 DM cases in the pandemic period. However, it was seen that previous studies on this subject were carried out using only the first-year data of the COVID-19 pandemic. The second-year data of the COVID-19 period in our study showed that the change in favor of type 2 DM in patients with DKA was temporary, and the rate of type 1 DM increased as the effect of the pandemic reduced.

In our study, it was shown that the mortality of diabetic emergency cases increased significantly in the COVID-19 period. The most important reason for this situation was that the deaths were directly related to COVID-19. This result was similar to most of the studies on this subject [6, 25-27].

In a retrospective study conducted in Mexico between 2017 and 2020 using the national death report system, it was found that diabetes-related mortality increased by 41.6% in 2020 compared to the average of the period between 2017 and 2019. In the same study, it was noted that there was an increase in the frequency of diabetes-related emergencies causing mortality. Compared to the 2018-2019 period, it was stated that a hyperglycemic hyperosmolar state increased by 128%, and diabetic ketoacidosis increased by 116% in 2020. These two conditions were the two most common causes of diabetes-related deaths [25].

Patel et al., in a study comparing the periods between March and May of 2019 and 2020, divided patients into three groups: patients with COVID-19 who developed DKA, patients without COVID-19 who developed DKA during the pandemic, and patients without COVID-19 who developed DKA in 2019 before the pandemic. The mortality rate for DKA patients with COVID-19 was 57%, for DKA patients without COVID-19 it was 2.1% (p=0.0001), and for diabetic patients (no DKA) with COVID-19 it was 39% (p=0.035) [26]. Similar to these findings, the main cause of mortality was found to be COVID-19 infection (60%) in our study.

Khan et al. reported that the prevalence of DKA increased from 0.72% to 3.14% in their study comparing March-April periods of 2019 and 2020. Additionally, they found out that DKA-related mortality also increased in 2020 (46.3% vs. 18%). In the same study, they suggested that more severe DKA did not increase the risk of death from COVID-19 [27].

In our study, no difference was found between the laboratory findings of individuals diagnosed with DKA during the COVID-19 pandemic and the pre-COVID-19 pandemic period. This finding was similar to the study by Misra et al. in 2021 [28].

There are only a few previous studies investigating the relationship between hypoglycemia and COVID-19. In one of these studies, it was mentioned that the risk of hypoglycemia increased, especially in type 2 DM patients who had comorbidities and were under sulfonylurea, insulin, and hydroxychloroquine treatment [29]. However, the increase in mortality due to hypoglycemia was not mentioned. Mesa et al., in their study with continuous glucose monitoring (CGM) of 92 patients with type 1 diabetes, stated that the frequency of hypoglycemia did not increase during the pandemic period [30]. In our study, 81.8% of the cases admitted to the emergency department with hypoglycemia were detected to be on sulfonylurea or insulin treatment. Like Shah et al., we also found that the frequency of hypoglycemia increased during the pandemic period [29], and in addition to that, we observed that COVID-19-related mortality increased in patients presenting with hypoglycemia.

Our analysis found that the upward trajectory in the number of endocrinology consultations prior to the pandemic continued to intensify during the pandemic. With the increase of diabetic patients all over the world, the number of endocrinology consultations requested due to diabetes-related emergency cases has gradually increased over the years. Since there is no study in the literature on this subject, no comparison regarding the rate of this increase could be made.

The limitation of our study is that it is a single-center and retrospective observational study; therefore, a causal relationship between the increased frequency of diabetes-related emergencies and COVID-19 cannot be established definitively. Our study provides a new perspective as it examines the process of diabetic emergencies in the general population rather than only COVID-19-positive patients. It is the first study to examine the relationship between COVID-19 and diabetes-related emergencies other than DKA together. In addition, our research indicated an increase in hypoglycemia frequency during the pandemic, and the related mortality rates may also have risen. Contrary to common knowledge, diabetic ketoacidosis was found to be more common in type 2 diabetics than in type 1 diabetics during the COVID-19 period.

## Conclusions

In summary, the findings of this study underscore the profound impact of the COVID-19 pandemic on diabetes management and the frequency of related emergencies. Our analysis reveals a significant increase in diabetic emergencies, particularly diabetic ketosis and hyperglycemic hyperosmolar state, during the pandemic period. This trend suggests that disruptions caused by the pandemic-such as reduced access to medical care, increased stress, and changes in lifestyle and treatment adherence- have compounded the challenges of managing diabetes effectively. Moreover, the notable rise in the number of type 2 diabetes patients experiencing diabetic ketoacidosis highlights the need for targeted interventions to support this subgroup, which may have been disproportionately affected by the pandemic's indirect effects.

It is important to learn the necessary lessons from this pandemic to better be prepared for future public health emergencies and ensure that patients with chronic diseases such as diabetes do not face increased risks due to systemic healthcare disruptions.
